# Efficacy of KNO_3_, SiO_2_ and SA priming for improving emergence, seedling growth and antioxidant enzymes of rice (*Oryza sativa*), under drought

**DOI:** 10.1038/s41598-021-83434-3

**Published:** 2021-02-16

**Authors:** Lawan Gana Ali, Rosimah Nulit, Mohd Hafiz Ibrahim, Christina Yong Seok Yien

**Affiliations:** 1 Department of Science Laboratory Technology, Mai Idris Alooma Polytechnic, Geidam, Yobe State Nigeria; 2 Department of Biology, Faculty of Science, Universiti Putra Malaysia, 43400 Selangor, Darul Ehsan Malaysia

**Keywords:** Physiology, Plant sciences

## Abstract

Rice is an important staple crop produced and consumed worldwide. However, poor seed emergence is one of the main impediments to obtaining higher yield of rice especially in hot and dry ecosystems of the world that are ravaged by drought. Therefore, this study was carried out to evaluate the effects of potassium nitrate (KNO_3_), salicylic acid (SA) and silicon dioxide (SiO_2_) priming in improving emergence, seedling growth, biochemical attributes and antioxidant activities of FARO44 rice under drought conditions. Rice seedlings primed with 2.5% and 5% KNO_3_, 3% and 3.5% SiO_2_, and 1 mM and 2.5 mM SA were subjected to three drought levels of low, moderate and severe under the greenhouse. Seed emergence, seedling growth, biochemical attributes and antioxidant activities were thereafter evaluated. Seed priming experiments were laid in a completely randomized design with five replicates per treatment. The results found that rice seedlings responded differently to different priming treatments. However, all primed rice seedlings had significantly (*P* ≤ 0.05) improved emergence percentage (72–92%), seedling growth, seedling vigor, seedling fresh and dry biomass and shorter emergence time compared with controls. Likewise, total soluble protein content, activities of catalase, ascorbate peroxidase and superoxide dismutase, carbohydrate, soluble sugar and total chlorophyll contents of rice seedlings were increased by more than two-folds by seed priming compared with control. Salicylic acid showed less effect in increasing emergence, seedling growth, antioxidant activities and biochemical attributes of rice. Thus, this study established that seed priming with KNO_3_ (2.5% and 5%) and SiO_2_ (3% and 3.5%) were more effective in improving emergence, seedling growth, biochemical attributes and antioxidant activities of FARO44. Thus, priming of FARO44 rice with this chemical is recommended for fast emergence, seedling growth and drought resistance in dry ecosystems.

## Introduction

Rice (*Oryza sativa* L.) is an important cereal that serves as a staple food for nearly 50% of the world’s population^1, 2^. Considering the importance of rice, there is the need for a stable and adequate supply of it for greater food security, poverty reduction and improvement of financial status of nations^3^. Several abiotic stresses affect emergence and growth of rice accounting for colossal yield loss and as such impede global rice production^2, 4^. Emergence of rice is affected by several environmental stresses that lead to poor yield. Poor emergence and seedling growth of rice caused by drought are serious yield reducing problems rice-growers faced particularly if seeds are broadcasted on dry soil^5, 6^. Drought causes poor emergence, growth and development of rice^7^. Severe drought causes total decline in the yield of rice^4^. Drought triggers several morphological, physiological and metabolic changes in plants^8^. It stimulates the generation of reactive oxygen species (ROS) in plants, ROS are very toxic and cause lipid peroxidation, destruction of protein, fragmentation of DNA, RNA, cell leakage, damage of cell membranes, destruction of photosynthetic constituents and finally death of cells^9^. These ROS consist of hydroxyl radicals (OH^−^), alkoxy radicals (RO^−^), superoxide radicals (O_2_^−^), perhydroxy radicals (HO_2_^−^), singlet oxygen (^1^O_2_) and hydrogen peroxide (H_2_O_2_)^8^. Rice responses to water deficit manifest in numerous morphological changes such as reduction of plant height, closing of stomata, decreased leaf elongation, low dry matter and leaf senescence^10^. To counter the effects of ROS, rice plants developed antioxidant enzymes such as superoxide dismutase (SOD), catalase (CAT), ascorbate peroxidase (APX) and glutathione peroxidases that scavenge excess ROS under stress conditions such as salinity, drought and extreme temperature^8^. Superoxide dismutase breaks down superoxide radical into H_2_O_2_ and H_2_O; CAT and APX break down H_2_O_2_ into H_2_O and O_2_. Glutathione peroxidases break down lipid hydroperoxides and H_2_O_2_ in plants^8^. There are many physiological approaches for improving emergence and drought tolerance of rice such as conventional breeding and selection, polyploidy breeding, mutation breeding and production of transgenic lines^3, 11^. However, these approaches are expensive, complicated and not easily accessible by low-skilled farmers^11, 12^. Priming of seeds is an easy, affordable and safe technique for enhancing emergence, seedling growth, yields and drought stress tolerance of crops^13^. It is a controlled hydration technique that stimulates pre-germination metabolic processes such as increased water imbibition, activation of reserve mobilizing amylase, cellulase and xylanase within seeds without actual germination^13, 14^. Priming of seeds has been shown to stimulate faster emergence rates, faster seedling establishment and vigorous seedling growth. Priming causes an increase in the activities of proteases, lipases and amylases that break down food reserves for enhanced embryonic growth and development^15^. Priming also minimises abiotic stress effect at the germination phase and finally leads to higher seedling emergence as well as vigorous seedling establishment. These biological activities of priming are useful for farmers because they decrease the emergence time, cost of repeated seeding, fertilization and additional irrigation^15^. Different methods of seed pre-soaking were used to stimulate essential pre-germination metabolic processes and seedling growth such as hydropriming, osmopriming, hormonal priming, biopriming, nutrient priming, solid matrix priming and magnetopriming^11, 16–19^. Different organic and inorganic chemicals, hormones and plant extracts were used for priming seeds^11, 25, 26^. However, several studies reported that silicon (Si), potassium nitrate (KNO_3_) and salicylic acid (SA) were effective priming agents that improve germination, growth, vigour and tolerance of wheat, maize, rice and cotton tolerance against drought, salinity and extreme temperatures^26–30^. Priming treatments with Si, KNO_3_ and SA were reported to improve emergence, seedling growth, yield, drought and salinity tolerance of rice, maize, wheat, and barley^20–24^. Seed priming with SiO_2_, KNO_3_ and SA were proven to improve germination, seedling growth, vigour and drought tolerance of rice, maize and wheat by stimulating increased water imbibition, activation of reserve mobilizing amylases, dehydrogenases, xylanase and variety of ROS-scavenging antioxidants^3, 17, 21, 22^. Seed priming with KNO_3_, Si, and SA improved proline, soluble protein, total soluble sugar and carbohydrate contents and decreased malondialdehyde content in rice, maize and wheat^3, 31, 32^. Moreover, KNO_3_, Si and SA priming of rice, maize and wheat substantially increased antioxidant activities of CAT, SOD and APX^3, 32, 33^.

Large part of Nigeria’s northern rice-growing areas were facing drought problems that led to poor emergence and seedling growth under a changing climate that ultimately affect yield. Even mild drought can inhibits floret initiation which causes spikelet sterility, grain filling and productivity decline^34^. Therefore, drought is a main problem that causes poor emergence and seedling growth of rice. Despite the effectiveness of seed priming in improving emergence, seedling growth and drought tolerance of rice, there is a dearth of seed priming study to address poor emergence and seedling growth of FARO44 rice. Therefore, this study aims to evaluate the efficacy of KNO_3_, SiO_2_ and SA priming on emergence, seedling growth, biochemical attributes and antioxidant enzyme activities of FARO44 rice grown under drought conditions.

## Results

### Seed priming enhanced emergence attributes of rice under drought

Seed priming with KNO_3_, SiO_2_ and SA had significant (*P* ≤ 0.05) effects on emergence percentage (EP), emergence index (EI) and mean emergence time (MET) of FARO44 rice as shown in Table 1. However, drought has no significant effect on EP and MET of FARO44 rice. Seed priming and its interaction with drought had significant effects on EP and EI of FARO44 rice. At mild drought, priming with 2.5% and 5% KNO_3_, 3% and 3.5% SiO_2_ and 1 mM and 2.5 mM SA increased EP of FARO44 rice by 70%, 94%, 94%, 84%, 96%, and 70% compared with control (54%). The highest EP were recorded by 1 mM SA primed FARO44 rice seedlings (96%), followed by 5% KNO_3_ (94%) and 3% SiO_2_ (94%) primed FARO44 rice seedlings. At moderate drought, priming with 2.5% and 5% KNO_3_, 3% and 3.5% SiO_2_ and 1 mM and 2.5 mM SA increased EP of FARO44 rice by 80%, 72%, 90%, 86%, 92% and 76% compared with control (32%). The highest EP were recorded by 1 mM SA (92%) and 3% SiO_2_ (90%) primed FARO44 rice seedlings. At severe drought, except 5% KNO_3_ and 3.5% SiO_2_, EP of FARO44 rice seedlings was increased by 2.5% KNO_3_, 3% SiO_2_, 1 mM and 2.5 mM priming by 90%, 80%, 90% and 86% compared with control (64%). The highest EP were recorded by 2.5% KNO_3_ (90%) and 1 mM SA (90%) primed FARO44 rice seedlings.

**Table 1:** Emergence attributes of KNO_3_, SiO_2_ and SA primed rice under drought conditions.

Drought levels	Priming treatments	EP (%)	EI	MET (days)
Mild	Control	54 ± 14.00^def^	0.74 ± 0.18^ fg^	7.8 ± 0.20^a^
	2.5% KNO_3_	70 ± 3.16^bcde^	1.24 ± 0.05^abcd^	7 ± 0.00^ab^
	5% KNO_3_	94 ± 2.00^ab^	1.44 ± 0.08^ab^	7 ± 0.00^ab^
	3% SiO_2_	94 ± 6.00^ab^	1.5 ± 0.10^a^	7 ± 0.00^ab^
	3.5% SiO_2_	84 ± 9.27^abc^	1.28 ± 0.13^abcd^	7 ± 0.00^ab^
	1 mM SA	96 ± 4.00^a^	1.46 ± 0.06^ab^	7 ± 0.00^ab^
	2.5 mM SA	70 ± 0.00^bcde^	1.12 ± 0.02^bcde^	7 ± 0.00^ab^
Moderate	Control	32 ± 11.57f.	0.46 ± 0.16^ g^	6 ± 1.51^b^
	2.5% KNO_3_	80 ± 7.07^abc^	1.20 ± 0.11^abcd^	7 ± 0.00^ab^
	5% KNO_3_	72 ± 7.34^abcd^	1.08 ± 0.05^cde^	7 ± 0.00^ab^
	3% SiO_2_	90 ± 6.32^ab^	1.38 ± 0.09^abc^	7 ± 0.00^ab^
	3.5% SiO_2_	86 ± 6.00^abc^	1.30 ± 0.08^abcd^	7 ± 0.00^ab^
	1 mM SA	92 ± 4.47^ab^	1.38 ± 0.08^abc^	7 ± 0.00^ab^
	2.5 mM SA	76 ± 5.09^abcd^	1.16 ± 0.08^abcde^	7 ± 0.00^ab^
Severe	Control	64 ± 4.00^cde^	0.96 ± 0.06^def^	7.6 ± 0.24^a^
	2.5% KNO_3_	90 ± 5.47^ab^	1.34 ± 0.06^abc^	7.2 ± 0.20^ab^
	5% KNO_3_	48 ± 10.67^ef^	0.74 ± 0.16^ fg^	7 ± 0.00^ab^
	3% SiO_2_	80 ± 8.36^abc^	1.20 ± 0.12^abcd^	7.2 ± 0.20^ab^
	3.5% SiO_2_	54 ± 6.78^def^	0.84 ± 0.11^ef^	7.2 ± 0.20^ab^
	1 mM SA	90 ± 3.74^ab^	1.38 ± 0.05^abc^	7 ± 0.00^ab^
	2.5 mM SA	86 ± 9.27^abc^	1.28 ± 0.12^abcd^	7.2 ± 0.20^ab^
Priming treatments		**	**	*
Drought		ns	*	ns
Priming × Drought		**	**	ns

Mean values ± SE in the same column followed with similar letters are not significantly different according to DMRT (*P* ≤ 0.05); *SE* Standard error of the mean, *EP* Emergence percentage, *EI* Emergence index, *MET* Mean emergence time, *Significant at 5% level of probability, **Significant at 1% level of probability, *ns* not significant.

At low mild drought, priming with 2.5% and 5% KNO_3_, 3% and 3.5% SiO_2_ and 1 mM and 2.5 mM SA increased EI of FARO44 rice by 1.24, 1.44, 1.5, 1.28, 1.46 and 1.12 compared with control (0.74). At moderate drought, priming with 2.5% and 5% KNO_3_, 3% and 3.5% SiO_2_ and 1 mM and 2.5 mM SA increased EI of FARO44 rice by 1.20, 1.08, 1.38, 1.30, 1.38 and 1.16 compared with control (0.46). At severe drought, except 5% KNO_3_ and 3.5% SiO_2_, EI of FARO44 was increased by 2.5% KNO_3_, 3% SiO_2_, 1 mM and 2.5 mM SA priming by 1.34, 1.20, 1.38 and 1.28 compared with control (0.96). At mild drought, KNO_3_, SiO_2_ and SA primed FARO44 rice seedlings showed shorter MET of 7 days compared with compared control (7.8 days). However, at moderate drought, KNO_3_, SiO_2_ and SA priming showed no significant effects on decreasing MET of rice compared with control. At severe drought, all KNO_3_, SiO_2_ and SA primed FARO44 rice seedlings had shorter MET compared with control. However, there were no significant differences in MET between different primed FARO44 rice seedlings.

### Seed priming improved seedling growth performance of rice under drought

Seed priming, drought and their interaction had significant (*P* ≤ 0.05) effects on seedling length, plumule and root length of FARO44 rice seedlings as presented in Table 2. At mild drought, all KNO_3_, SiO_2_ and SA priming treatments increased seedling length of FARO44 rice compared with control. FARO44 rice seedlings primed with 3% and 3.5% SiO_2_ recorded the highest seedling length (50.95 cm and 51.48 cm). At moderate drought, seedling length of FARO44 rice was significantly enhanced by KNO_3_, SiO_2_ and SA priming compared with control (unprimed). FARO44 rice seedlings primed with 5% KNO_3_ and 3.5% SiO_2_ recorded the highest seedling length of 52.54 cm and 49.11 cm. At severe drought, except 3.5% SiO_2_, all primed FARO44 rice seedlings were significantly longer than control. FARO44 rice seedlings primed with 2.5% KNO_3_ and 1 mM SA recorded the longest seedlings of 41.35 cm and 43.3 cm.

**Table 2:** Seedling growth performance of rice primed with KNO_3_, SiO_2_ and SA under drought conditions.

Drought levels	Priming treatments	Seedling length (cm)	Plumule length (cm)	Root length (cm)
Mild	Control	31.06 ± 0.72^j^	24.08 ± 0.62^ h^	6.43 ± 0.25^gh^
	2.5% KNO_3_	46.02 ± 1.00^bcdef^	36.96 ± 0.91^bcde^	8.86 ± 0.42^bc^
	5% KNO_3_	48.38 ± 1.82^abcd^	39.48 ± 1.41^ab^	9.22 ± 0.43^ab^
	3% SiO_2_	50.95 ± 2.01^ab^	41.88 ± 1.62^a^	8.72 ± 0.46^bc^
	3.5% SiO_2_	51.48 ± 2.45^ab^	42.98 ± 2.07^a^	8.45 ± 0.45^bcde^
	1 mM SA	44.54 ± 0.76^cdefg^	37.06 ± 0.62^bcde^	7.50 ± 0.29^cdefg^
	2.5 mM SA	37.18 ± 1.51i	27.91 ± 1.26^gh^	9.19 ± 0.37^ab^
Moderate	Control	15.09 ± 2.64^ k^	11.79 ± 2.07^ij^	3.27 ± 0.58^i^
	2.5% KNO_3_	42.68 ± 1.35^efghi^	34.14 ± 1.16^def^	8.49 ± 0.35^bcd^
	5% KNO_3_	52.54 ± 1.93^a^	42.22 ± 1.59^a^	10.26 ± 0.42^a^
	3% SiO_2_	47.04 ± 1.90^abcde^	38.49 ± 1.61^abcd^	8.38 ± 0.41^bcde^
	3.5% SiO_2_	49.11 ± 1.85^abc^	39.35 ± 1.53^abc^	8.03 ± 0.30^bcdef^
	1 mM SA	41.18 ± 1.03^fghi^	33.23 ± 0.88^ef^	7.98 ± 0.12^bcdef^
	2.5 mM SA	43.58 ± 1.31^cdefg^	34.79 ± 1.34^cdef^	8.33 ± 0.30^bcdef^
Severe	Control	12.99 ± 1.40^ k^	8.74 ± 1.03^j^	4.37 ± 0.51^i^
	2.5% KNO_3_	41.35 ± 1.46^fghi^	34.19 ± 1.16^def^	7.20 ± 0.40^defgh^
	5% KNO_3_	31.53 ± 3.21^j^	24.69 ± 2.56^ h^	6.98 ± 0.75^fgh^
	3% SiO_2_	37.91 ± 1.80^hi^	31.94 ± 1.54^ fg^	6.1 ± 0.34^ h^
	3.5% SiO_2_	18.83 ± 1.59^ k^	14.19 ± 1.21^i^	4.58 ± 0.48^i^
	1 mM SA	43.33 ± 0.90^defgh^	35.68 ± 0.99^bcdef^	7.31 ± 0.32^defgh^
	2.5 mM SA	39.63 ± 1.78^ghi^	32.45 ± 1.54^ef^	7.06 ± 0.39^efgh^
Priming treatments		**	**	**
Drought		**	**	**
Priming × Drought		**	**	**

Mean values ± SE in the same column followed with similar letters are not significantly different according to DMRT (*P* ≤ 0.05); *SE* Standard error of the mean, *Significant at 5% level of probability, **Significant at 1% level of probability, *ns* Not significant.

Under all drought levels, plumule length of FARO44 rice seedlings was increased by KNO_3_, SiO_2_ and SA priming compared with control. Moreover, KNO_3_, SiO_2_ and SA primed FARO44 rice seedlings recorded significantly increased root length compared with control under all drought levels. However, under severe drought, 3.5% SiO_2_ priming showed no significant effect on plumule length of rice seedlings compared with control.

### Seed priming enhanced seedling biomass and vigor of rice under drought

Seed priming, drought and their interaction had significant (*P* ≤ 0.05) effects on seedling fresh and dry biomass, seedling vigor index I (SVI I) and seedling vigor index II (SVI II) of FARO44 rice as presented in Table 3. At mild drought, KNO_3_ and 3% SiO_2_ primed FARO44 rice seedlings had significantly higher seedling fresh biomass compared with control. However, 3.5% SiO_2_ and SA priming had no significant effects on fresh biomass of FARO44 rice seedlings compared with control. The maximum fresh biomass (630 mg) was recorded by 5% KNO_3_ primed rice seedlings under mild drought. At moderate drought, all primed FARO44 rice seedlings had improved fresh biomass compared with control. FARO44 rice seedlings primed with 5% KNO_3_ (408 mg) had the highest fresh biomass under moderate drought. At severe drought, except 3.5% SiO_2_, all other priming treatments significantly increased fresh biomass of FARO44 rice seedlings compared with control. Maximum fresh biomass (347.97 mg) was recorded by 2.5% KNO_3_ primed FARO44 rice seedlings under severe drought. At mild drought, except 1 mM SA, dry biomass of FARO44 rice seedlings was enhanced by all priming treatments compared with control. FARO44 rice seedlings primed with 3% SiO_2_ had the highest fresh biomass of 90.84 mg compared with other primed rice seedlings. At moderate and severe drought, dry biomass of FARO44 rice seedlings was significantly improved by KNO_3_, SiO_2_ and SA priming compared with control. However, 3.5% SiO_2_ priming had no significant effect on dry biomass of FARO44 rice seedlings at severe drought compared with control.

**Table 3:** Effects of KNO_3_, SiO_2_ and SA priming on seedling biomass and vigour of rice under drought conditions.

Drought levels	Priming treatments	Seedling fresh biomass (mg)	Seedling dry biomass (mg)	SVI I	SVI II
Mild	Control	316 ± 12.03^de^	54.22 ± 1.37^ef^	1993 ± 11.01f.	3459.2 ± 19.15^gh^
	2.5% KNO_3_	373.99 ± 9.87^bc^	59.06 ± 1.76^de^	3208.53 ± 35.26^d^	4047.2 ± 20.17^ef^
	5% KNO_3_	630 ± 18.87^a^	62.48 ± 2.27^ cd^	4735.5 ± 40.06^a^	6094.2 ± 32.44^bc^
	3% SiO_2_	390 ± 7.62^b^	90.84 ± 1.99^a^	4693.57 ± 35.13^a^	8469.67 ± 50.71^a^
	3.5% SiO_2_	324.07 ± 12.59^de^	75.67 ± 2.73^b^	4189.93 ± 20.15^b^	6391.3 ± 30.09^b^
	1 mM SA	277.93 ± 1.41f.	50.00 ± 1.32^ fg^	4252.33 ± 30.03^b^	4765 ± 33.04^d^
	2.5 mM SA	288 ± 10.00^ef^	58.82 ± 1.35^de^	2594.43 ± 13.02^e^	4117.4 ± 11.03^ef^
Moderate	Control	94.47 ± 16.50^i^	22.49 ± 3.99^j^	763.9 ± 11.20^ h^	1083.63 ± 11.01^j^
	2.5% KNO_3_	385.13 ± 15.99^b^	46.49 ± 1.60^gh^	3338.87 ± 24.11^d^	3617.3 ± 14.03^ g^
	5% KNO_3_	408 ± 16.25^b^	63.46 ± 1.84^ cd^	3672.67 ± 33.74^c^	4434.6 ± 47.75^de^
	3% SiO_2_	342 ± 7.09^ cd^	64.90 ± 1.40^ cd^	4120.3 ± 18.02^b^	5763.87 ± 24.09^c^
	3.5% SiO_2_	314 ± 12.84^de^	67.43 ± 1.46^c^	4123.87 ± 24.32^b^	5708.13 ± 48.09^c^
	1 mM SA	266.6 ± 4.97^ fg^	42.26 ± 0.34^ h^	3680.87 ± 13.04^c^	3790.97 ± 8.12^ fg^
	2.5 mM SA	236 ± 3.28^gh^	41.42 ± 0.78^ h^	3247.97 ± 38.39^d^	3106.93 ± 9.06^hi^
Severe	Control	116.20 ± 12.91^i^	16.75 ± 1.83^j^	871.40 ± 12.04^ h^	1090.9 ± 10.02^j^
	2.5% KNO_3_	347.97 ± 12.74^ cd^	48.48 ± 0.74^ fg^	3644.2 ± 52.82^c^	4327.43 ± 26.65^e^
	5% KNO_3_	266.20 ± 22.72^ fg^	31.37 ± 2.96^i^	1481.2 ± 15.99^ g^	1506.77 ± 7.12^j^
	3% SiO_2_	222.00 ± 7.28^ h^	47.02 ± 2.75^gh^	2884.77 ± 12.07^e^	3517.47 ± 31.03^ g^
	3.5% SiO_2_	127.25 ± 9.85^i^	19.92 ± 1.78^j^	924.93 ± 6.02^ h^	1101.53 ± 10.01^j^
	1 mM SA	231.99 ± 11.11^gh^	32.14 ± 1.18^i^	3939.57 ± 24.0^bc^	2892.47 ± 15.03^i^
	2.5 mM SA	203.13 ± 8.53^ h^	31.79 ± 1.22^i^	3321.2 ± 31.12^d^	2750.07 ± 13.06^i^
Priming treatments		**	**	**	**
Drought		**	**	**	**
Priming × Drought		**	**	**	**

Mean values ± SE in the same column followed with similar letters are not significantly different according to DMRT (*P* ≤ 0.05); *SE* Standard error of the mean, *SVI I* Seedling vigour index I, *SVI II* Seedling vigour index II, *Significant at 5% level of probability, **Significant at 1% level of probability, *ns* Not significant.

Across all drought levels, KNO_3_, SiO_2_ and SA priming enhanced SVI I of FARO44 rice compared with control. However, at severe drought, 3.5% SiO_2_ primed rice seedlings were not significantly different from control. At mild and moderate drought, SVI II of FARO44 rice seedlings were significantly improved by KNO_3_, SiO_2_ and SA priming compared with control. At severe drought, KNO_3_, SiO_2_ and SA priming improved SVI II of FARO44 rice seedlings compared with control. However, 5% KNO_3_ and 3.5% SiO_2_ primed rice seedlings were not significantly different from control.

### Priming improved total soluble protein content, catalase, ascorbate peroxidase and superoxide dismutase activities of rice seedlings under drought

Seed priming had significant (*P* ≤ 0.05) effects on total soluble protein content, catalase (CAT), ascorbate peroxidase (APX) and superoxide dismutase (SOD) activities of FARO44 rice seedlings. However, drought and its interaction with priming had significant effects on total soluble protein content of FARO44 rice seedlings, but not on CAT, APX and SOD activities of FARO44 rice seedlings as shown in Table 4. Except 1 mM SA primed rice seedlings under moderate drought, total soluble protein content of FARO44 rice seedlings was enhanced by priming treatments under all drought levels compared with control. Under all drought levels, KNO_3_, SiO_2_ and SA priming increased CAT activities of FARO44 rice seedlings compared with control. Priming with 3.5% SiO_2_ showed more effect in increasing CAT activities of FARO44 rice seedlings than other priming treatments. At mild drought, only SiO_2_ primed FARO44 rice seedlings had higher APX activities, KNO_3_ and SA priming showed no significant effects in increasing APX activities of rice seedlings compared with control. At moderate drought, except 5% KNO_3_ and 2.5 mM SA primed rice seedlings, other primed FARO44 rice seedlings had significantly increased APX activities compared with control. At severe drought, only FARO44 rice seedlings primed with 3% and 3.5% SiO_2_ and 1 mM SA had significantly increased APX activities, other primed rice seedlings were not significantly different from control. Under all drought levels, except KNO_3_, SOD activities of rice seedlings were significantly increased by SiO_2_ and SA priming. Priming with SA showed more effect in increasing SOD activities of FARO44 rice seedlings than other priming treatments.

**Table 4:** Total soluble protein content, CAT, APX and SOD activities of rice seedlings primed with KNO_3_, SiO_2_ and SA under drought conditions.

Drought levels	Priming treatments	Total soluble protein (mg/µg^−1^FW)	CAT activity (U/mg^−1^protein)	APX activity (U/mg^−1^protein)	SOD activity (U/mg^−1^FW)
Mild	Control	0.87 ± 0.02^d^	0.02 ± 0.01^ fg^	0.26 ± 0.07^b^	0.18 ± 0.06^cde^
	2.5% KNO_3_	1.33 ± 0.03^a^	0.03 ± 0.00^efg^	0.27 ± 0.06^b^	0.07 ± 0.01^e^
	5% KNO_3_	1.24 ± 0.02^ab^	0.06 ± 0.02^cdef^	0.34 ± 0.10^b^	0.11 ± 0.02^e^
	3% SiO_2_	1.19 ± 0.10^b^	0.07 ± 0.01^bcde^	0.41 ± 0.07^ab^	0.34 ± 0.07^b^
	3.5% SiO_2_	1.28 ± 0.02^ab^	0.11 ± 0.02^ab^	0.87 ± 0.17^a^	0.26 ± 0.05^bcd^
	1 mM SA	1.06 ± 0.02^c^	0.06 ± 0.01^bcdef^	0.25 ± 0.04^b^	0.58 ± 0.03^a^
	2.5 mM SA	1.06 ± 0.02^c^	0.07 ± 0.01^abcde^	0.27 ± 0.04^b^	0.52 ± 0.03^a^
Moderate	Control	0.99 ± 0.02^c^	0.01 ± 0.00^ g^	0.26 ± 0.08^b^	0.25 ± 0.05^bcd^
	2.5% KNO_3_	1.33 ± 0.04^a^	0.05 ± 0.01^defg^	0.36 ± 0.08^ab^	0.09 ± 0.02^e^
	5% KNO_3_	1.21 ± 0.01^b^	0.08 ± 0.01^abcd^	0.25 ± 0.04^b^	0.05 ± 0.01^e^
	3% SiO_2_	1.29 ± 0.02^ab^	0.10 ± 0.01^abc^	0.69 ± 0.13^ab^	0.34 ± 0.03^b^
	3.5% SiO_2_	1.26 ± 0.01^ab^	0.12 ± 0.03^a^	0.60 ± 0.19^ab^	0.32 ± 0.07^b^
	1 mM SA	1.04 ± 0.02^c^	0.08 ± 0.01^abcd^	0.69 ± 0.40^ab^	0.53 ± 0.04^a^
	2.5 mM SA	1.22 ± 0.05^ab^	0.06 ± 0.01^bcdef^	0.20 ± 0.03^b^	0.49 ± 0.02^a^
Severe	Control	0.36 ± 0.03f.	0.00 ± 0.00^ g^	0.21 ± 0.07^b^	0.15 ± 0.03^de^
	2.5% KNO_3_	0.83 ± 0.02^de^	0.05 ± 0.01^defg^	0.23 ± 0.04^b^	0.06 ± 0.02^e^
	5% KNO_3_	0.83 ± 0.02^de^	0.08 ± 0.01^abcd^	0.24 ± 0.05^b^	0.04 ± 0.01^e^
	3% SiO_2_	0.77 ± 0.01^e^	0.10 ± 0.01^abc^	0.67 ± 0.13^ab^	0.29 ± 0.04^bc^
	3.5% SiO_2_	0.74 ± 0.03^e^	0.10 ± 0.02^abc^	0.61 ± 0.19^ab^	0.28 ± 0.07^bc^
	1 mM SA	0.73 ± 0.04^e^	0.07 ± 0.01^abcde^	0.70 ± 0.40^ab^	0.52 ± 0.03^a^
	2.5 mM SA	0.74 ± 0.04^e^	0.06 ± 0.01^cdef^	0.20 ± 0.03^b^	0.47 ± 0.02^a^
Priming treatments		**	**	**	**
Drought		**	ns	ns	ns
Priming × Drought		**	ns	ns	ns

Mean values ± SE in the same column followed with similar letters are not significantly different according to DMRT (*P* ≤ 0.05); *SE* Standard error of the mean, *CAT* Catalase, *APX* Ascorbate peroxidase, *SOD* Superoxide dismutase, *Significant at 5% level of probability, **Significant at 1% level of probability, *ns* Not significant.

### Seed priming enhanced carbohydrate, total soluble sugar, total chlorophyll contents and decreased malondialdehyde content of rice seedlings under drought

Seed priming, drought and their interaction had significant (*P* ≤ 0.05) effects on carbohydrate, total soluble sugar, total chlorophyll and malondialdehyde contents of FARO44 rice seedlings as shown in Table 5. Under all drought levels, except SiO_2_ priming, carbohydrate content of FARO44 rice seedlings was significantly improved by KNO_3_ and SA priming compared with control. At mild drought, except 3.5% SiO_2_, total soluble sugar content of FARO44 rice seedlings was significantly enhanced by 2.5% and 5% KNO_3_, 3% SiO_2_ and 1 mM and 2.5 mM SA priming compared with control. At moderate drought, KNO_3_, SiO_2_ and SA significantly increased total soluble sugar content of FARO44 rice seedlings compared with control. At severe drought, except SA, total soluble sugar content of FARO44 rice seedlings was markedly increased by KNO_3_ and SiO_2_ priming compared with control. Priming with KNO_3_ showed more effect in increasing total soluble sugar content of FARO44 rice seedlings than other priming treatments. Under all drought levels, total chlorophyll content of FARO44 rice seedlings was significantly increased by KNO_3_, SiO_2_ and SA priming compared to non-primed rice seedlings. Under all drought levels, malondialdehyde (MDA) content of FARO44 rice seedlings was significantly decreased by KNO_3_, SiO_2_ and SA priming compared with control. Malondialdehyde causes lipid peroxidation and oxidative stress in rice seedlings.

**Table 5:** Effects of KNO_3_, SiO_2_ and SA priming on carbohydrate, total soluble sugar, chlorophyll and MDA contents of rice seedlings under drought conditions.

Drought levels	Priming treatments	Carbohydrate (mg g^−1^FW)	Total soluble sugar (mg g^−1^FW)	Total chlorophyll (mg g^−1^FW)	MDA (µmol/mg^−1^FW)
Mild	Control	1.71 ± 0.03^b^	1.54 ± 0.04^de^	17.33 ± 1.05^ij^	0.025 ± 0.01^b^
	2.5% KNO_3_	3.68 ± 0.05^a^	2.91 ± 0.08^a^	25.05 ± 0.52^cdef^	0.001 ± 0.00^c^
	5% KNO_3_	3.72 ± 0.08^a^	2.73 ± 0.02^a^	24.97 ± 1.50^cdef^	0.001 ± 0.00^c^
	3% SiO_2_	1.67 ± 0.11^b^	2.42 ± 0.19^b^	23.37 ± 1.78^efg^	0.001 ± 0.00^c^
	3.5% SiO_2_	1.73 ± 0.11^b^	1.51 ± 0.08^e^	24.53 ± 1.48^def^	0.001 ± 0.00^c^
	1 mM SA	3.66 ± 0.13^a^	2.34 ± 0.08^b^	28.79 ± 1.77^bc^	0.005 ± 0.00^c^
	2.5 mM SA	3.59 ± 0.04^a^	2.43 ± 0.17^b^	32.87 ± 1.12^a^	0.001 ± 0.00^c^
Moderate	Control	1.71 ± 0.01^b^	1.35 ± 0.05^ef^	17.69 ± 1.17^ij^	0.072 ± 0.01^a^
	2.5% KNO_3_	3.74 ± 0.09^a^	2.73 ± 0.12^a^	20.34 ± 1.20^ghi^	0.001 ± 0.00^c^
	5% KNO_3_	3.78 ± 0.10^a^	2.88 ± 0.09^a^	27.99 ± 1.70^bcd^	0.001 ± 0.00^c^
	3% SiO_2_	1.69 ± 0.10^b^	1.97 ± 0.12^c^	21.95 ± 1.43^fgh^	0.001 ± 0.00^c^
	3.5% SiO_2_	1.73 ± 0.03^b^	1.79 ± 0.06^ cd^	26.79 ± 2.32^cde^	0.001 ± 0.00^c^
	1 mM SA	3.59 ± 0.06^a^	2.07 ± 0.09^c^	26.19 ± 1.21^cde^	0.002 ± 0.00^c^
	2.5 mM SA	3.63 ± 0.06^a^	2.79 ± 0.14^a^	31.42 ± 0.88^ab^	0.027 ± 0.01^b^
Severe	Control	1.09 ± 0.02^c^	1.17 ± 0.05f.	16.04 ± 0.65^j^	0.080 ± 0.01^a^
	2.5% KNO_3_	1.70 ± 0.10^b^	1.96 ± 0.11^c^	17.16 ± 0.64^ij^	0.001 ± 0.00^c^
	5% KNO_3_	1.72 ± 0.11^b^	1.87 ± 0.09^c^	16.97 ± 0.30^ij^	0.001 ± 0.00^c^
	3% SiO_2_	1.19 ± 0.02^c^	1.35 ± 0.05^ef^	18.01 ± 0.05^ij^	0.002 ± 0.00^c^
	3.5% SiO_2_	1.14 ± 0.01^c^	1.47 ± 0.03^ef^	18.39 ± 1.13^hij^	0.001 ± 0.00^c^
	1 mM SA	1.56 ± 0.06^b^	1.17 ± 0.02f.	16.44 ± 0.49^ij^	0.023 ± 0.01^b^
	2.5 mM SA	1.59 ± 0.05^b^	1.19 ± 0.02f.	16.38 ± 0.64^ij^	0.024 ± 0.01^b^
Priming treatments		**	**	**	**
Drought		**	**	**	**
Priming × Drought		**	**	**	**

Mean values ± SE in the same column followed with similar letters are not significantly different according to DMRT (*P* ≤ 0.05); *SE* Standard error of the mean, *DMRT *Duncan’s multiple range test, *Significant at 5% level of probability, **Significant at 1% level of probability, *ns* Not significant, *MDA* Malondialdehyde.

## Discussion

This study investigated the influence of KNO_3_, SiO_2_ and SA seed priming to improve emergence, seedling growth, biochemical attributes and antioxidant activities of rice seedlings under drought. It is well known that rice production in arid and semi-arid ecosystems of the world are being ravaged by drought under a changing climate. Drought affects germination, seedling emergence and yield of rice^35^. This study found that KNO_3_, SiO_2_ and SA priming substantially improved emergence percentage, emergence index and decreased mean emergence time of FARO44 rice under drought. Improved emergence attributes of rice might be related to increased cell division and elongations, water imbibition by seeds, repair and synthesis of DNA and RNA, increased activities of reserve mobilizing enzymes such as acid phosphatase, dehydrogenase, α-amylase and β-amylase in primed seeds. Primed rice seedlings had increased activities of catalase, ascorbate peroxidase and superoxide dismutase activities and low lipid peroxidation which are important factors in plant for deficit water resistance. Priming of seeds stimulate pre-germination processes in rice, wheat and maize that improved faster germination and seedling emergence^36^. Many germination processes such as increased water imbibition, cell division and hydrolytic enzymes in rice were stimulated by priming which triggered faster germination as well as establishment of seedlings under stressful conditions^37,38^. Previous results by Yuan-yuan et al.^39^, found that rice priming with water and polyethylene glycol under drought had considerably increased emergence percent, emergence index and decreased emergence time. Zhang et al.^9^ reported that sorghum primed with polyethylene glycol grown under drought showed improved emergence percentage, emergence index and vigour index. Khaliq et al.^40^ found that rice priming with selenium substantially increased emergence, emergence index and decreased mean emergence time. Tabatabaei et al.^41^ reported that SA priming of sorghum substantially increased percent emergence, emergence index and decreased emergence time under drought.

This study found that KNO_3_, SiO_2_ and SA priming substantially increased seedling growth of rice under drought. Enhancement of rice seedling growth might be due to increased cell division and elongation and activation of ROS scavenging enzymes in primed seeds. Potassium nitrate, silicon dioxide and SA stimulated rice seedling growth by serving as nutrients and initiators of essential emergence and growth processes in seedlings. Previously, Zhang et al.^9^ found that sorghum priming with polyethylene glycol increased shoot length, root length and seedling length under drought. Khaliq et al.^40^ reported that selenium priming of rice considerably improved shoot and root length, and seedling dry biomass. Tabassum et al.^22^ found that wheat priming with CaCl_2_ and hydropriming enhanced plant height, leaf area, panicle length and grain yield under terminal drought.

Seedling biomass and vigour of FARO44 rice seedlings grown under drought were improved by KNO_3_, SiO_2_ and SA priming. Improved biomass and vigour of rice were as a results of increased cell elongation and division, nucleic acid synthesis and repair in primed seeds. Similar results were reported by Farooq et al.^19^ that SA priming of rice improved growth, seedling fresh and dry weights under drought. Selenium primed rice and PEG primed sorghum showed substantially enhanced seedling dry biomass and vigor index under drought ^9,40^. Javed et al.^5^ reported that two rice cultivars primed with 0.75% KNO_3_ recorded significantly increased seedling growth, seedling biomass and vigour. Previously, Wang et al.^38^ reported that salicylic acid and selenium primed rice seedling under chilling stress recorded increased germination, shoot and root fresh biomass, shoot and root length.

The present study found that total soluble protein content, antioxidant activities of catalase, ascorbate peroxidase and superoxide dismutase of FARO44 rice seedlings were improved by KNO_3_, SiO_2_ and SA priming under drought. Improvement of protein content and antioxidant activities in primed rice seedlings indicated enhanced tolerance to drought stress. Increased antioxidant activities in rice seedlings justified the increased seedling emergence and seedling growth of rice under drought. Increased protein content and antioxidant activities in rice seedlings were associated with the potentials of KNO_3_, SiO_2_ and SA priming for activating antioxidant machinery, synthesis of protein and nucleic acids. Under water deficit conditions, plants developed antioxidant machinery that scavenge excess ROS which impair lipid, protein and nucleic acids thereby causing lipid peroxidation and oxidative stress^42^. Under drought, plants accumulate soluble solutes and increase activities of antioxidants that scavenge ROS that cause oxidative stress and lipid peroxidation^35^. Corroborating these findings, Ahmad et al.^43^ reported that maize priming with salicylic acid and ascorbic acid improved catalase and peroxidase activities with consequent increase in germination, shoot and root fresh weight and dry weight under drought. Higher activities of these enzymes in primed maize seedlings under drought suggested their roles in minimizing harmful effects of drought on plant growth and development. Abdel-Latef and Tran^21^ reported that silicon priming of maize under alkalinity stress markedly recorded increased in total soluble protein, CAT, POD and SOD activities. Agreeing these results, earlier study has found that rice primed with salicylic acid and ascorbic acid under drought stress exhibited increased activities of CAT and APX^41^. Previously, Zhang et al.^9^ reported that PEG primed sorghum grown under drought stress showed substantially improved CAT, SOD, APX and POD activities. Rice seedlings primed with selenium and SA under drought stress were found to have enhanced total soluble protein, GPX (glutathione peroxidase), CAT, APX, and SOD activities^19,40^. Hussain et al.^44^ reported that two rice cultivars primed with SA and Se grown under low temperature stress recorded substantially higher activities of CAT, POD, SOD and glutathione.

This study found that priming with KNO_3_, SiO_2_ and SA substantially enhanced carbohydrate, total soluble sugar and total chlorophyll contents and decreased malondialdehyde content in FARO44 rice seedlings under drought. Enhanced carbohydrate, soluble sugar and total chlorophyll contents in rice seedlings were essential for osmotic adjustment and increased photosynthetic activities under drought conditions. Increased carbohydrate, soluble sugar and total chlorophyll contents in rice seedlings justified their increased emergence, growth and seedling vigour under drought. Low malondialdehyde content in primed rice seedlings suggested defense from lipid peroxidation and oxidative stress. Active accumulation of soluble solutes such as carbohydrate, soluble sugar, glycine betaine and proline is a key tolerance strategy shown by plants under drought^45^. Corroborating these results, Zhang et al.^9^ found that sorghum primed with PEG grown under drought stress has significantly higher total soluble sugar and chlorophyll contents and reduced malondialdehyde content. Previous results of Parveen et al.^33^ found that two maize varieties primed with 4 mM and 6 mM Si recorded increased emergence, seedling growth, activities of POD, SOD, CAT and APX, and low malondialdehyde content and increased content of glycine betaine, proline and soluble sugar. Khaliq et al.^40^ reported that rice seedlings primed with selenium had markedly improved soluble sugar and total chlorophyll content. Abdel-Latef and Tran^21^ found that Si primed maize grown under alkalinity stress showed substantially increased total chlorophyll and soluble sugar contents. Wang et al.^38^ reported that rice seedlings primed with Se and SA under chilling stress showed higher total soluble sugar content. Jisha and Puthur^46^ found that three rice cultivars osmoprimed with NaCl and grown under drought and salt stresses had increased carbohydrate and total chlorophyll contents.

## Conclusions

Priming with concentrations of 2.5% and 5% KNO_3_, 3% and 3.5% SiO_2_ and 1 mM and 2.5 mM SA responded differently in improving emergence, seedling growth, biochemical attributes and antioxidant activities of FARO44 rice seedlings grown under drought conditions. Increased emergence, seedling growth, biochemical attributes and antioxidant activities of rice seedlings indicated increased tolerance to drought. Seed pre-soaking with 2.5% and 5% KNO_3_ and 3% and 3.5% SiO_2_ were found to be more effective in improving emergence, seedling growth, biochemical attributes and antioxidant enzyme activities of rice seedlings than SA priming. Increased emergence and seedling growth of primed rice might be associated with the potentials of KNO_3_ and SiO_2_ priming in stimulating pre-germination metabolic events such as increased water imbibition, cell division and elongation, repair of damaged nucleic acids, activation of reserve mobilizing enzymes and antioxidant machinery within the seeds that eventually enhanced emergence, growth and vigour. The findings of this study justified the reliability of seed priming, an easy and affordable technique to be adopted by farmers in dry regions of the world for improving emergence, seedling establishment and growth under drought conditions.

## Methods

FARO44 rice seeds (*Oryza sativa* L.) were obtained from Badeggi Rice Research Institute in north-central Nigeria. It is a rice variety with a long grain produced from a hybridization between Taiwan Indica and African local rice^47^. It is high yielding and matures in 110–120 days. It can be produced under rain-fed and irrigation agriculture^47,48^. The seed initial moisture level was 10.9% while the dried seed moisture was 8.97% on the basis of dry weight.

### Pre-optimization of priming chemical concentrations and duration

To obtain reliable results, preliminary priming studies were performed by soaking rice seeds in varying concentrations of KNO_3_, SiO_2_ and SA with different durations prior to obtaining the effective priming duration (8 h) and priming chemical concentrations^44^. The effective concentrations used for this study are: KNO_3_ (2.5% and 5% w/v), SiO_2_ (3% and 3.5%) and SA (1 mM and 2.5 mM). These selections were on the bases of germination and seedling growth performances.

### Seed priming treatments

Prior to seed pre-soaking treatments, the different concentrations of SiO_2_, KNO_3_ and SA were prepared and kept in a fridge. Viable quality rice seeds were surface sterilized in 0.5% sodium hypochlorite (v/v) for 10 min to suppresses microbial growth and rinsed thoroughly with distilled water. Rice seeds were separately soaked in solutions of KNO_3_ (2.5% and 5% w/v), SiO_2_ (3% and 3.5% w/v) and SA (1 mM and 2.5 mM) for 8 h, and the systems were kept in the dark laboratory growth room at 25 ± 2 °C, relative humidity of 50–70% and a photoperiod of 12 h light/12 h dark. The ratio of 1:5 (w/v) seed weight to solution volume was maintained^49^. The seeds were dried back to their near-original weight of 10.1% at 25 °C temperature for 48 h prior to germination tests^50^.

### Rice emergence and drought stress induction experiment

The emergence experiments were carried out in the Greenhouse of the Department of Biology, University Putra Malaysia (3.000384° N, 101.705545° E), Selangor. To assess the emergence and seedling growth of rice under drought conditions, pot experiments were conducted. Plastic pots (24 cm × 20 cm) were filled with 2 kg silty-loamy soil. The soil used for the experiment had organic matter content (0.94%), saturation percentage (0.94%), electrical conductivity (1.02 dSm^−1^), total nitrogen content (0.07%), potassium content (167.00 ppm), phosphorus content (6.00 ppm) and chloride ion content (8.30 mmol L^−1^). Ten (n = 10) rice seeds primed with 2% and 5% KNO_3_; 3% and 3.5% SiO_2_; 1 mM and 2.5 mM SA and unprimed seeds (control) were separately sown in each of the pots. After sowing the seeds, the pots were watered daily and maintained at 100% field capacity (FC) (well-watered) for thirty days prior to imposition of drought stress. Three levels of drought stress were imposed to the rice seedlings by limited watering, these include: mild drought (75% FC), moderate drought (50% FC) and severe drought (25% FC) for twenty-one days^2,4^. All priming experiments were laid in a completely randomized design (CRD) with five replicates. All the pots were placed in the greenhouse with 12:12 h light/night duration, day and night temperatures of 35 °C and 27 °C. Records of seed emergence were taken daily until all seeds emerged, a seed was considered emerged if the radicle was about 2 mm long^51^. Seedlings were harvested after 51 days and the following emergence and seedling growth parameters from six randomly selected seedlings from each replicates were measured^52^:

$${\text{Emergence}}\;{\text{percentage}}\;{\text{was}}\;{\text{computed}}\;{\text{using}}\;{\text{EP}} = \frac{No\;of\;seeds\;normally\;emerged}{{Total\;no.\;of\;seeds\;emerged}} \times 100.$$

Emergence index defined as the total number of seeds that emerge daily was calculated as^53^:

$${\text{EI}} = \frac{No\;of\;emerged\;seeds}{{Days\;of\;first\;count - No. \;of\;seeds\;emerged/day \;of\;final\;count}}.$$

Mean emergence time (MET) was evaluated using ${\text{MET}} = \frac{\Sigma Dn}{{\Sigma {\text{n}}}}$, where n stands for the number of seeds that emerged on day D and D is the number of days counted from the start of emergence^54^.

Seedling length, shoot length and root length were measured with a ruler^52^.

Fresh seedling biomass was weighed with an electronic balance while the dry seedling biomass was weighed after drying at 80 °C in an oven for 48 h^55^.

Seedling vigor index I (SVI I) was calculated with SVI I = Seedling length × emergence percentage ^52^.

Seedling vigor II was calculated with SVI II = Seedling dry biomass × emergence percentage^52^.

### Biochemical analyses and Antioxidant enzymes assays

Total soluble protein content of KNO_3_, SiO_2_ and SA primed rice seedlings was determined according to the slightly modified method of Bradford^56^, while Bovine serum albumin (BSA) was used as a standard.

Catalase activity of KNO_3_, SiO_2_ and SA primed rice seedlings was evaluated according to the method of Zhang et al.^9^. The activity of CAT was expressed in U/mg^−1^protein. One unit of CAT activity was defined as the change in absorbance of 0.01 units per minute.

Ascorbate peroxidase activity of KNO_3_, SiO_2_ and SA primed rice seedlings was evaluated according to the method described by Nakano and Asada^57^ by determining the absorbance decrease of oxidized ascorbate after every 15 s for 1 min at wavelength of 290 nm (ε = 2.8 mM^−1^ cm^−1^). The activity of APX was expressed in U/mg^−1^protein.

Superoxide dismutase activity of KNO_3_, SiO_2_ and SA primed rice seedlings was determined according to the method described by Zheng et al.^3^. The decrease of NBT was evaluated by reading the absorbance change at 560 nm with a spectrophotometer (Model-Hitachi U-1900, Tokyo, Japan), and the activity of SOD was expressed as U/mg^−1^FW.

Extraction and determination of total chlorophyll content of KNO_3_, SiO_2_ and SA primed rice seedlings were performed according to the method described by Lichtenthaler and Wellburn^58^. The total chlorophyll content was calculated with the formula: Total Chlorophyll = C_a_ + C_b_ + C_x + c_. C_a_ = 13.95(A_665_) − 6.88(A_649_); C_b_ = 24.96(A_649_) − 7.32(A_665_); C_x + c_ = (1000A_470_ − 2.05C_a_ − 114.8C_b_)/245; Where, C_a_ = chlorophyll a, C_b_ = chlorophyll b and C_x+c_ = carotenoid.

Total carbohydrate content of KNO_3_, SiO_2_ and SA primed rice seedlings was determined according to the method described by Nielsen^59^. Total carbohydrate content was determined from a standard curve prepared with mg/ml of D-glucose solution.

Total soluble sugar of KNO_3_, SiO_2_ and SA primed rice seedlings was determined according to the method described by Watanabe^60^. The total soluble sugar content was calculated from a linear equation based on a standard curve produced from d-glucose. The absorbance of the reaction mixture was read at 620 nm with a spectrophotometer (Model-Hitachi U-1900, Tokyo, Japan).

Lipid peroxidation of KNO_3_, SiO_2_ and SA primed rice seedlings was determined by referring to the malondialdehyde (MDA) content and was measured according to the slightly modified method of^61^. First, fresh leaf sample weighing 250 mg was frozen in liquid nitrogen. The frozen leaves were ground in chilled mortar placed on ice in 3 millilitre of trichloroacetic acid (TCA). The mixture was then centrifuged at 13,000 g for 7 min. at 4 °C. The supernatant (2 ml) was mixed with 2 ml of 0.67% thiobarbituric acid. The mixture was then heated in a water-bath set at 100 °C for 30 min. and the reaction was swiftly terminated by cooling in ice. The mixture was centrifuged again for 12,000 g for 10 min. for the suspended particles to settle. The absorbance of the supernatant collected was read at 532 and 600 nm. The mixture of 0.025% thiobarbituric acid in 10% trichloroacetic acid was used as a blank sample. The content of MDA (µmolmg^−1^ FW) was computed with the formula: MDA = (A532-A600)/ε, ε is the extinction coefficient (155 mM^−1^ cm^−1^).

### Statistical analyses

Prior to statistical analyses, normality of all the data was checked with the Kolmogorov–Smirnov test. Two-way analyses of variance were performed to compare the effects of seed priming and drought on emergence, seedling growth, biochemical attributes and antioxidant activities of FARO44 rice (ANOVA) with an SPSS (window version 24). Significant differences of means were separated using the Duncan's Multiple Range test (*P* ≤ 0.05)^55^.

## Data Availability

The data supporting the findings of this study will be obtained from the corresponding author upon request.
